# Paternal Split‐Liver Transplantation Followed by Haploidentical Hematopoietic Cell Transplantation in an Adult Patient With Protoporphyria‐Induced Liver Failure

**DOI:** 10.1002/jha2.1092

**Published:** 2025-02-18

**Authors:** Ulrich Stölzel, Lasse Jost, Daniel Seehofer, Katharina Egger‐Heidrich, Uwe Scheuermann, Kristina Hölig, Thomas Stauch, Desiree Kunadt, Detlef Schuppan, Johannes Schetelig, Nils Wohmann, Martin Bornhäuser, Friedrich Stölzel

**Affiliations:** ^1^ Department of Internal Medicine II and Porphyria Center Klinikum Chemnitz Chemnitz Germany; ^2^ Division of Stem Cell Transplantation and Cellular Immunotherapies Department of Internal Medicine II University Hospital Schleswig Holstein Kiel, Kiel University Kiel Germany; ^3^ Department of Visceral Transplantation, Vascular and Thoracic Surgery University Hospital of Leipzig Leipzig Germany; ^4^ Department of Internal Medicine I University Hospital Carl Gustav Carus, TU Dresden Dresden Germany; ^5^ Deutsches Kompetenz‐Zentrum für Porphyriediagnostik und Konsultation MVZ Labor Volkmann und Kollegen GbR Karlsruhe Germany; ^6^ Institute of Translational Immunology Johannes Gutenberg University Mainz Mainz Germany; ^7^ Division of Gastroenterology Beth Israel Deaconess Medical Center Harvard Medical School Boston Massachusetts USA

**Keywords:** haploidentical, liver disease, porphyria, stem cell transplantation

## Abstract

**Introduction:**

Erythropoietic Protoporphyria (EPP) caused skin light sensitivity and liver cirrhosis in a 35‐year‐old patient who subsequently developed liver‐failure.

**Methods:**

In absence of a human leukocyte antigens (HLA)‐matched‐unrelated donor, the father consented in donating for split liver transplantation (SLT) and allogeneic hematopoietic cell transplantation (HCT).

**Results:**

After bridging therapy and successful SLT a first paternal HCT resulted in graft failure. For a second haploidentical HCT a different regimen was applied leading to engraftment while protoporphyrin (PP) blood‐levels decreased to normal and skin light sensitivity skin disappeared, leading to complete remission in an immunosuppressive‐free patient.

**Conclusion:**

Haploidentical transplantation is a feasible treatment approach in EPP‐patients.

**Trial Registration:**

The authors have confirmed clinical trial registration is not needed for this submission

**Abbreviations**
aGvHD: acute graft‐versus‐host disease
ALA: aminolevulinic acid
ALT: alanine aminotransferase
AST: aspartate aminotransferase
ATG: anti‐thymocyte globulin
BID: bis in die (twice a day)
BMT: bone marrow transplantation
CD: cluster of differentiation
COVID‐19: coronavirus disease 2019
DSAs: donor‐specific HLA‐antibodies
EPP: erythropoietic protoporphyria
FECH: ferrochelatase
G‐CSF: granulocyte‐colony stimulating factor
GGT: gamma glutamyltransferase
HCT: hematopoietic cell transplantation
HLA: human leukocyte antigens
ISP: immunosuppressive prophylaxis
IST: immunosuppressive therapy
IVIG: intravenous‐immunoglobulin
MMF: mycophenolate‐mofetil
OMIM: Online Mendelian Inheritance in Man
PBG: porphobilinogen
PGF: primary graft failure
PP: protoporphyrin
PTCy: post‐transplant cyclophosphamide
SAA: severe aplastic anemia
SLT: split‐liver transplantation
TAC: tacrolimus

## 1 Introduction

**FIGURE 1:**
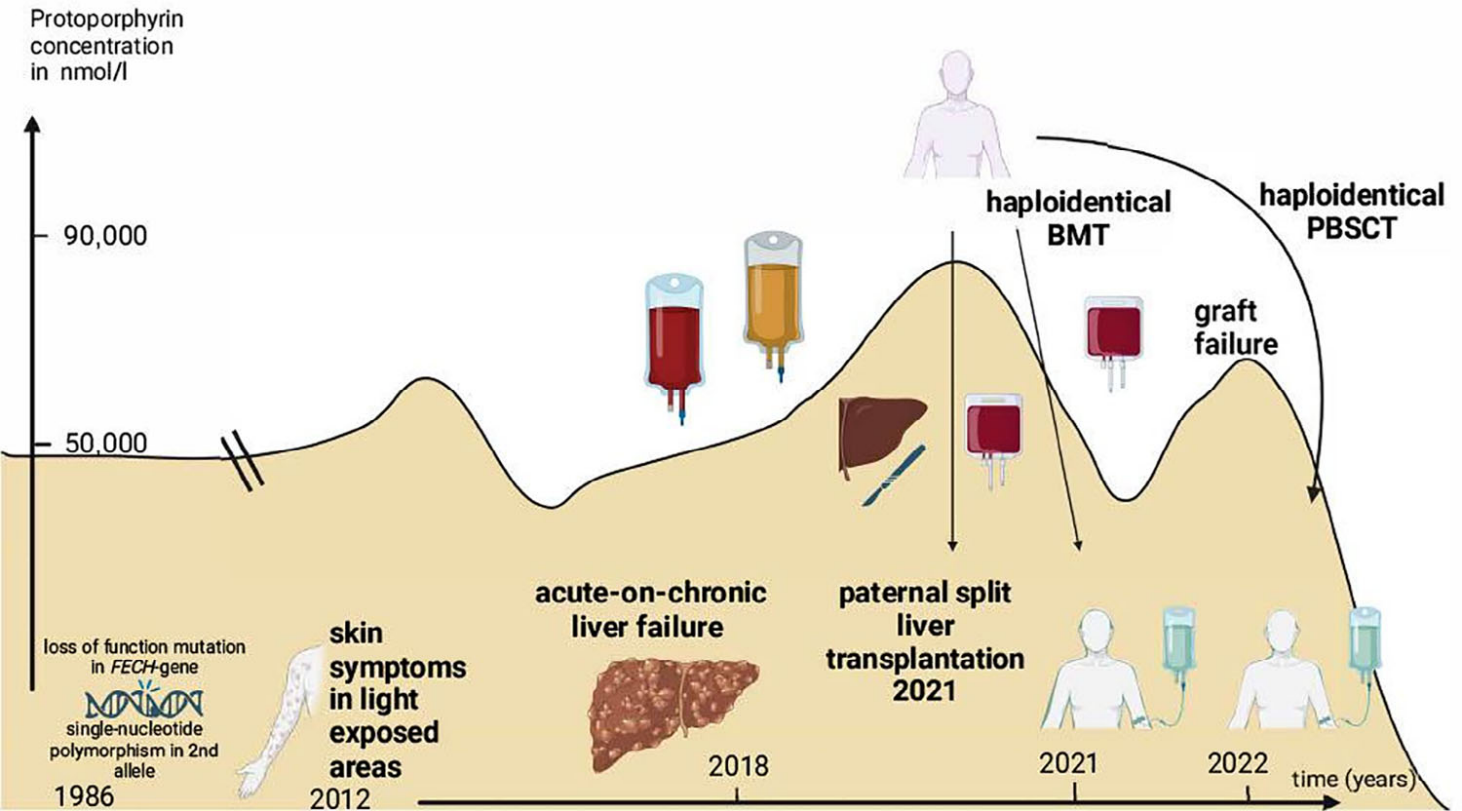
Visualization of the disease course of the patient from birth until latest follow‐up after the second allogeneic, haploidentical hematopoietic cell transplantation (HCT). Chronological time on the x‐axis, protoporphyrin concentration on the y‐axis. Left liver‐lobe and scalpel represent liver living donor split transplantation from the haploidentical father. Large red‐ and yellow‐colored transfusion bags display repeated erythrocyte‐apheresis and plasmapheresis, respectively. Small red transfusion bags display haploidentical hematopoietic cell transplantation from the father of the patient.

At the age of two years a child was presented with redness, pain, and swelling on sunlight exposed skin areas (Figure 1). Diagnosis of erythropoietic protoporphyria (EPP) was established based on increased erythrocyte protoporphyrin IX (PP) concentrations (Online Mendelian Inheritance in Man (OMIM) reference 177000) [1, 2, 3].

In 1993, when he was 6 years old, erythrocyte PP concentration was 38,032 nmol/L (normal < 89%). The proportion of metal free vs. zinc PP was 99% vs. 1%, respectively. At that time, he did not display clinical or laboratory signs of liver damage.

Porphyrin precursors aminolevulinic acid (ALA) and porphobilinogen (PBG), and porphyrins in urine were within normal range while fecal excretion of PP was increased (300 µg/g). Interestingly, at the time of diagnosis the proportion of urinary coproporphyrin isomers I vs III was increased up to 77% (normal < 31%) indicating early hepato‐biliary dysfunction. Consistent with the diagnosis of EPP, analysis of the *ferrochelatase* (*FECH*) gene detected a heterozygous loss of function mutation c.973delA, p.(Arg325Glyfs*11), and a low‐expression IVS3‐48C variant [4].

### 1.1 Patient Pre‐Transplant Treatment and Outcome

Therapy comprised thorough light protection, vitamin D substitution, oral beta‐carotene in the first years, later cholestyramine, and time shifted ursodeoxycholic acid. Despite microcytic anemia (MCV 77.7 fl, normal >80%; hemoglobin 7.9 mmol/L, normal > 8.6%), iron substitution was avoided, since ferritin concentration and transferrin saturation were within normal range. Furthermore, iron may activate PP synthesis potentially aggravating the disease course in patients with EPP [5].

The patient reported low quality of life and restriction of almost all of his outdoor activities during day time. To attenuate light sensitivity afamelanotide, an alpha‐melanocyte‐stimulating agent, was subcutaneously implanted from spring to autumn beginning at the age of 29 years [6].

**FIGURE 2:**
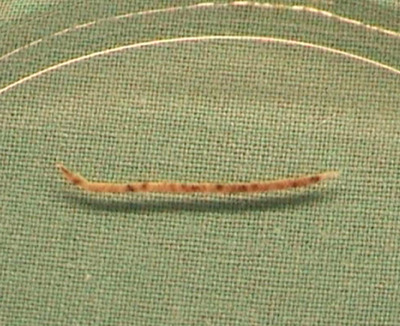
Liver biopsy demonstrated blackish pigmented cylinder.

**FIGURE 3:**
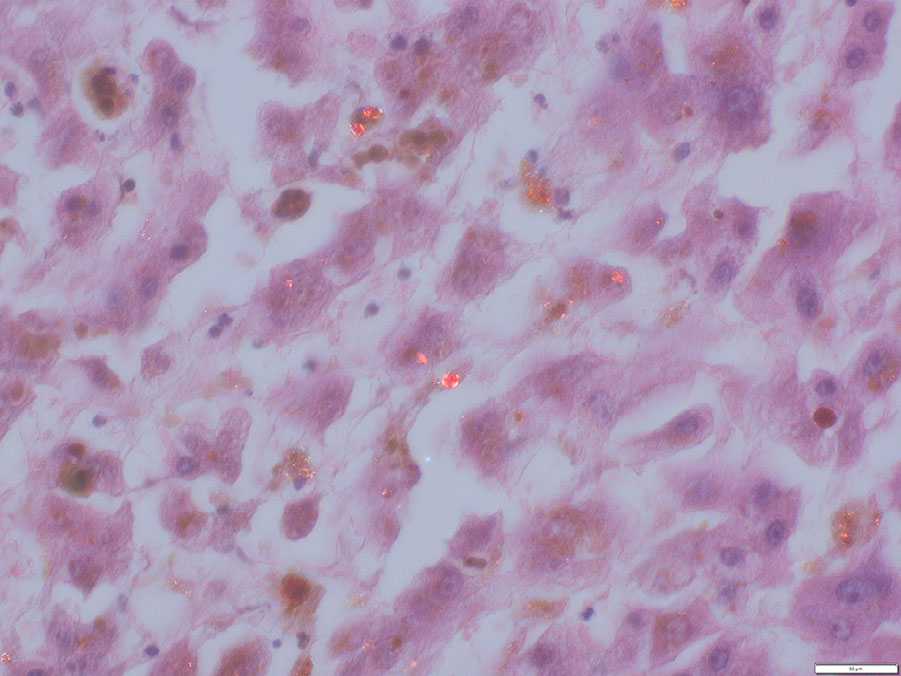
Liver biopsy with broad septa, canalicular cholestasis predominantly in zone 1, and spherical structures displaying red birefringence under polarized light with a dark ”Maltese cross” in the center (original magnification × 400). The histological pictures were kindly provided by Hendrik Bläker, Institute of Pathology, University of Leipzig Medical Center.

While liver enzyme activities from the first decade of his life were normal, at the age of 26 years, mild‐to‐moderate increase of alanine aminotransferase (ALT) 2.7 µkat/L, aspartate aminotransferase (AST) 1.6 µkat/L, and gamma glutamyltransferase (GGT) 3.9 µkat/L were observed (normal < 0.85%, < 0.62%, < 1.0%, respectively). Liver biopsies demonstrated blackish pigmented cylinders (Figure 2) with microscopically pre‐cirrhotic stage 3, later cirrhotic stage 4 (Figure 3). At stage 4 liver cirrhosis, abdominal ultrasound and shear wave elastography demonstrated hepato‐splenomegaly with liver stiffness of 14.3 kPa (cut‐off for cirrhosis > 11 kPa).

At this time allogeneic hematopoietic cell transplantation (HCT) as the only curative treatment option available was considered and an unrelated donor search was initiated, resulting in non‐availability of a human leukocyte antigens (HLA)‐matched‐unrelated donor [7]. Therefore, the haploidentical, healthy father in whom EPP was excluded by laboratory and genetic testing, was identified as the best available hematopoietic stem cell donor. However, the intention to perform HCT before occurrence of liver failure was postponed due to the beginning of the coronavirus disease 2019 (Covid‐19) pandemic.

### 1.2 Sequential Solid Organ and Haploidentical Allogeneic Transplantation

At the age of 35 years and still in the Covid‐19 pandemic, the patient developed severe liver failure. He suffered from right upper abdominal pain and jaundice (bilirubin 149 µmol/L, ALT 5.6 µkat/L, AST 4.9 µkat/L, GGT 3.8 µkat/L) peaking in erythrocyte PP of 92,245 nmol/L (normal < 89%) in August 2020. Liver stiffness increased up to 48.8 kPa. Extrahepatic cholestasis and other causes of liver damage were excluded. Consequently, repeated plasmapheresis and subsequent erythrocyte‐apheresis were initiated in an alternating manner (Figure 1) [8]. This regimen led to a transient reduction in PP in the peripheral blood to 46,246 nmol/L, reduction of liver enzyme activities, and relief of symptoms. Due to the acuity of liver decompensation and successful re‐compensation by apheresis support, the patient was scheduled for living donor split liver‐transplantation (SLT) from his haploidentical father [9]. SLT under yellow‐filter protection during surgery was performed successfully and the early post‐SLT recovery went uneventful. Although immunosuppressive prophylaxis (ISP) was performed with tacrolimus (TAC) and mycophenolate‐mofetil (MMF), rejection of the transplanted allograft was suspected which led to initiation of immunosuppressive therapy (IST) with immunoadsorption and prednisolone‐therapy. Under IST, symptoms and laboratory parameters indicating graft‐rejection alleviated and planned haploidentical HCT from the same haploidentical donor could be performed. However, due to a bile‐duct variant, percutaneous bile drainage from segment 6 was still necessary throughout the haploidentical HCT. Due to the existence of donor‐specific HLA‐antibodies (DSAs), the patient underwent plasmapheresis, treatment with rituximab and intravenous‐immunoglobulin (IVIG) administration and conditioning with fludarabine (150 mg/m^2^ i.v.) and treosulfan (38 g/m^2^ i.v.) before receiving the haploidentical bone marrow graft with subsequent ISP using post‐transplant cyclophosphamide (PTCy, 2 × 50 mg/kg i.v.), TAC, and MMF [10]. Although the patient initially engrafted with increasing peripheral blood counts and increasing donor chimerism, he eventually experienced non‐DSA‐associated primary graft failure (PGF) with subsequent autologous reconstitution at Day +54. The allogeneic haploidentical bone marrow transplantation (BMT)‐induced decrease of PP had a trough at 2,056 nmol/L but then steadily increased again (Figure 1). As pre‐conditioning regimen before a second haploidentical HCT, the patient then received hydroxyurea (500 mg p.o. BID) which led to a moderate reduction in PP levels and gradually improved peripheral blood counts. Due to the previous PGF, the patient underwent a total‐body irradiation (TBI)‐based preparative conditioning regimen using fludarabine (150 mg/m^2^ i.v.), cyclophosphamide (29 mg/kg i.v.), 4 Gy total body irradiation, and rabbit anti‐thymocyte globulin (ATG) (4.5 mg/kg i.v.) before undergoing HCT using peripheral blood stem cells (6.4 × 10^6^ CD34^+^/kg) from the same donor 9 months after the first haploidentical BMT. Using a regimen initially established for patients with severe aplastic anemia (SAA) undergoing HCT including the same post‐transplantation ISP with PTCy, TAC, and MMF, the patient had a complete donor‐chimerism on Day +21, engraftment of neutrophils on Day +29 and platelets on Day +35 without granulocyte‐colony stimulating factor (G‐CSF) or further transfusion support [11]. An episode of grade 2 lower‐GI‐acute graft‐versus‐host disease (aGvHD) and grade 1 skin aGvHD was treated with 2 mg/kg prednisolone under which all symptoms alleviated instantly. Subsequently, PP levels decreased and normalized by Day +236 after second HCT. A further episode of suspected skin aGvHD grade 2 was treated with prednisolone and topical treatment. Subsequently, MMF was terminated on Day +145 after second HCT, and tapering of TAC was initiated on Day +246 and was completed recently in the absence of aGvHD. Due to the secondary immunodeficiency after HCT the patient had to be readmitted several times to treat recurrent infectious complications including multi‐resistant gram‐negative bacteria which are reported to occur frequently in patients after liver transplantation. Biliary complications like duct leakage and/or constriction occur in 20% of cases after right lobe living donor SLT [12]. Currently, at Day +793 the successful second paternal haploidentical HCT led to disappearance of the light sensitivity. During this time the patient reported having visited his hometown maypole festival for the first time in his life during daytime.

## 2 Discussion

Successful sequential liver transplantation and HLA‐matched HCT in pediatric and adult patients with EPP have been reported [13]. One 17 year‐old EPP patient was likewise treated with SLT and haploidentical HCT, both from his father. Since PGF occurred, the procedure was not curative [14]. Recently, SLT followed by haploidentical HCT was reported successfully for the first time in a pediatric patient with EPP [15]. Here, we report the first case of paternal SLT followed by haploidentical HCT with PTCy and the first case where these sequential transplantations were performed in an adult patient with protoporphyria‐induced liver failure. Thus, demonstrating additional therapeutic options for clinicians currently discussed in guidelines for management of protoporphyria‐induced liver failure [8].

## 3 Conclusion

Due to the advances in haploidentical transplantation, even in adult patients with acute‐ on chronic liver failure sequential haploidentical SLT and HCT can be performed successfully.

## Author Contributions

All authors participated in writing the paper as well as in the treatment of the patient mentioned in the case description either in a treating capacity or in consulting capacity.

## Conflicts of Interest

The authors declare no conflicts of interest.

## Data Availability

The data that support the findings of this study are available from the corresponding author upon reasonable request.

## References

[1] H. Puy , L. Gouya , and J.‐C. Deybach , “Porphyrias,” Lancet 375, no. 9718 (2010): 924–937. 20226990 10.1016/S0140-6736(09)61925-5

[2] D. M. Bissell , K. E. Anderson , and H. L. Bonkovsky , “Porphyria,” New England Journal of Medicine 377, no. 9 (2017): 862–872. 28854095 10.1056/NEJMra1608634

[3] U. Stölzel , M. O. Doss , and D. Schuppan , “Clinical Guide and Update on Porphyrias,” Gastroenterology 157, no. 2 (2019): 365–381. 31085196 10.1053/j.gastro.2019.04.050

[4] L. Gouya , H. Puy , A.‐M. Robreau , et al., “The Penetrance of Dominant Erythropoietic Protoporphyria Is Modulated by Expression of Wildtype FECH,” Nature Genetics 30, no. 1 (2002): 27–28. 11753383 10.1038/ng809

[5] J. Barman‐Aksoezen , X. Schneider‐Yin , and E. Minder , “Iron in Erythropoietic Protoporphyrias: Dr. Jekyll or Mr. Hyde?,” Journal of Rare Diseases Research & Treatment 2, no. 4 (2017): 1–5.

[6] J. G. Langendonk , M. Balwani , K. E. Anderson , et al., “Afamelanotide for Erythropoietic Protoporphyria,” New England Journal of Medicine 373, no. 1 (2015): 48–59. 26132941 10.1056/NEJMoa1411481 PMC4780255

[7] S. Wahlin , J. Aschan , M. Björnstedt , U. Broomé , and P. Harper , “Curative Bone Marrow Transplantation in Erythropoietic Protoporphyria After Reversal of Severe Cholestasis,” Journal of Hepatology 46, no. 1 (2007): 174–179. 17112627 10.1016/j.jhep.2006.10.004

[8] C. Levy , A. K. Dickey , B. Wang , et al., “Evidence‐Based Consensus Guidelines for the Diagnosis and Management of Protoporphyria‐Related Liver Dysfunction in Erythropoietic Protoporphyria and X‐Linked Protoporphyria,” Hepatology 79, no. 3 (2024): 731–743. 37505211 10.1097/HEP.0000000000000546 PMC10818013

[9] Z. S. Ardalan , S. Chandran , A. Vasudevan , et al., “Management of Patients With Erythropoietic Protoporphyria‐Related Progressive Liver Disease,” Liver Transplantation 25, no. 11 (2019): 1620–1633. 31469227 10.1002/lt.25632

[10] S. O. Ciurea , M. M. Al Malki , P. Kongtim , et al., “Treatment of Allosensitized Patients Receiving Allogeneic Transplantation,” Blood Advances 5, no. 20 (2021): 4031–4043. 34474478 10.1182/bloodadvances.2021004862 PMC8945639

[11] A. E. DeZern , M. L. Zahurak , H. J. Symons , et al., “Haploidentical BMT for Severe Aplastic Anemia With Intensive GVHD Prophylaxis Including Posttransplant Cyclophosphamide,” Blood Advances 4, no. 8 (2020): 1770–1779. 32343796 10.1182/bloodadvances.2020001729 PMC7189283

[12] K. S. H. Chok , S. C. Chan , T. T. Cheung , et al., “Bile Duct Anastomotic Stricture After Adult‐to‐Adult Right Lobe Living Donor Liver Transplantation,” Liver Transplantation 17, no. 1 (2011): 47–52. 21254344 10.1002/lt.22188

[13] A. L. Windon , R. Tondon , N. Singh , et al., “Erythropoietic Protoporphyria in an Adult With Sequential Liver and Hematopoietic Stem Cell Transplantation: A Case Report,” American Journal of Transplantation 18, no. 3 (2018): 745–749. 29116687 10.1111/ajt.14581

[14] C. Frieri , A. Poli , M. Balsat , and F. S. de Fontbrune , “The Role of Allogeneic Stem Cell Transplantation in Severe Erythropoietic Protoporphyria in Adults and Young Adults: Timing and Modalities,” Hematology, Transfusion and Cell Therapy 46, no. Supplement 5 (2024): S294–S298. 38719715 10.1016/j.htct.2024.02.027 PMC11670562

[15] S. Malkiel , B. A. Sayed , V. Ng , et al., “Sequential Paternal Haploidentical Donor Liver and HSCT in EPP Allow Discontinuation of Immunosuppression Post‐Organ Transplant,” Pediatric Transplantation 25, no. 6 (2021): e14040. 34076929 10.1111/petr.14040

